# Supplementary figures and images for: Genetic Diversity of Epichloë Endophytes Associated with Brachypodium and Calamagrostis Host Grass Genera including Two New Species
Source: J Fungi (Basel). 2022 Oct 15;8(10):1086. doi: 10.3390/jof8101086 (PMC9605649; doi:10.3390/jof8101086)

**Suppl. S2.** Principal Coordinates (PCoA) of asexual isolates from *B. sylvaticum*

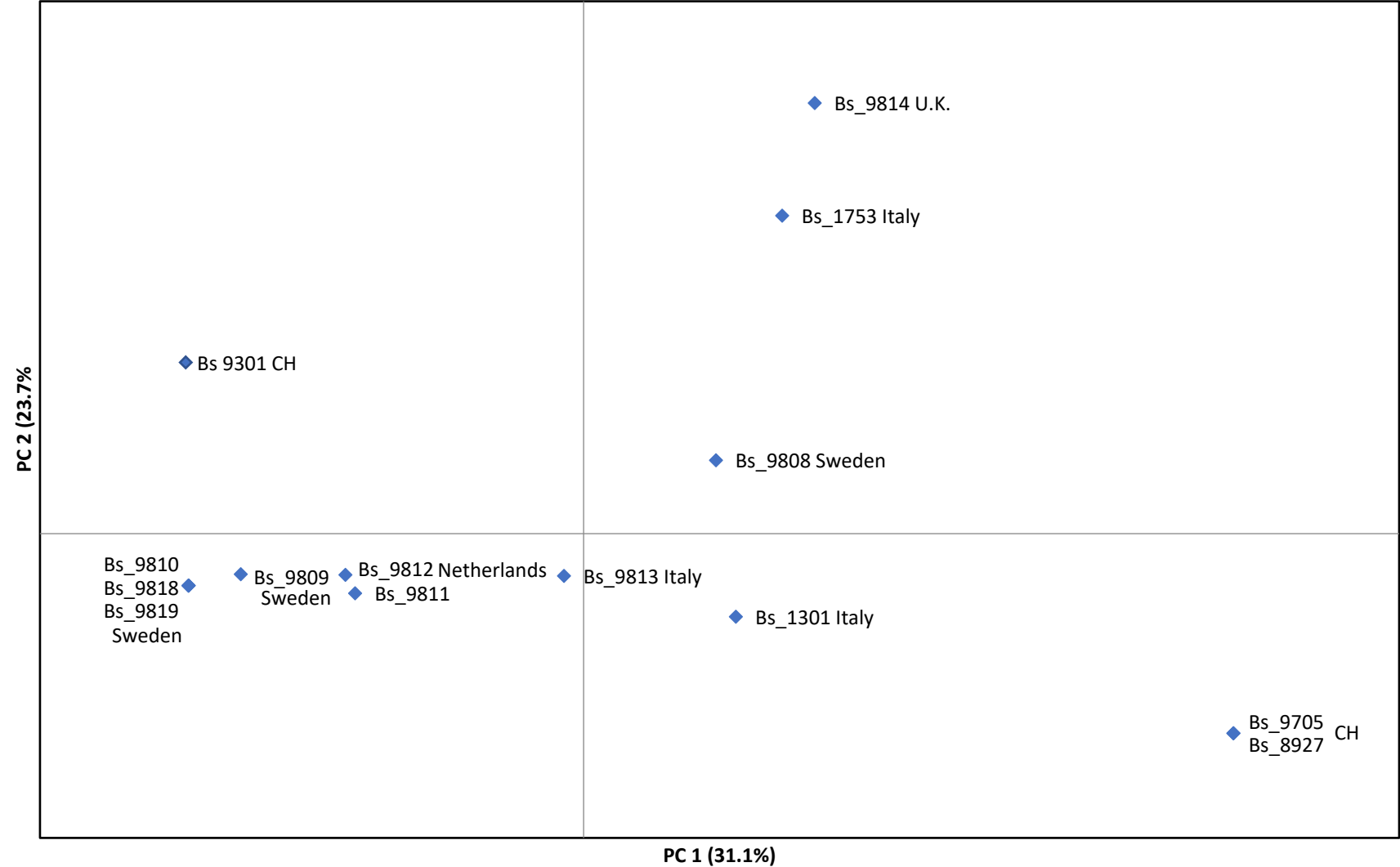

Supplement: Supplementary file 1 [file jof-08-01086-s001.zip › Suppl. S2.pdf]

**Suppl. S3.** Principal Coordinates (PCoA) of isolates from *B. pinnatum* and *B. phoenicoides*

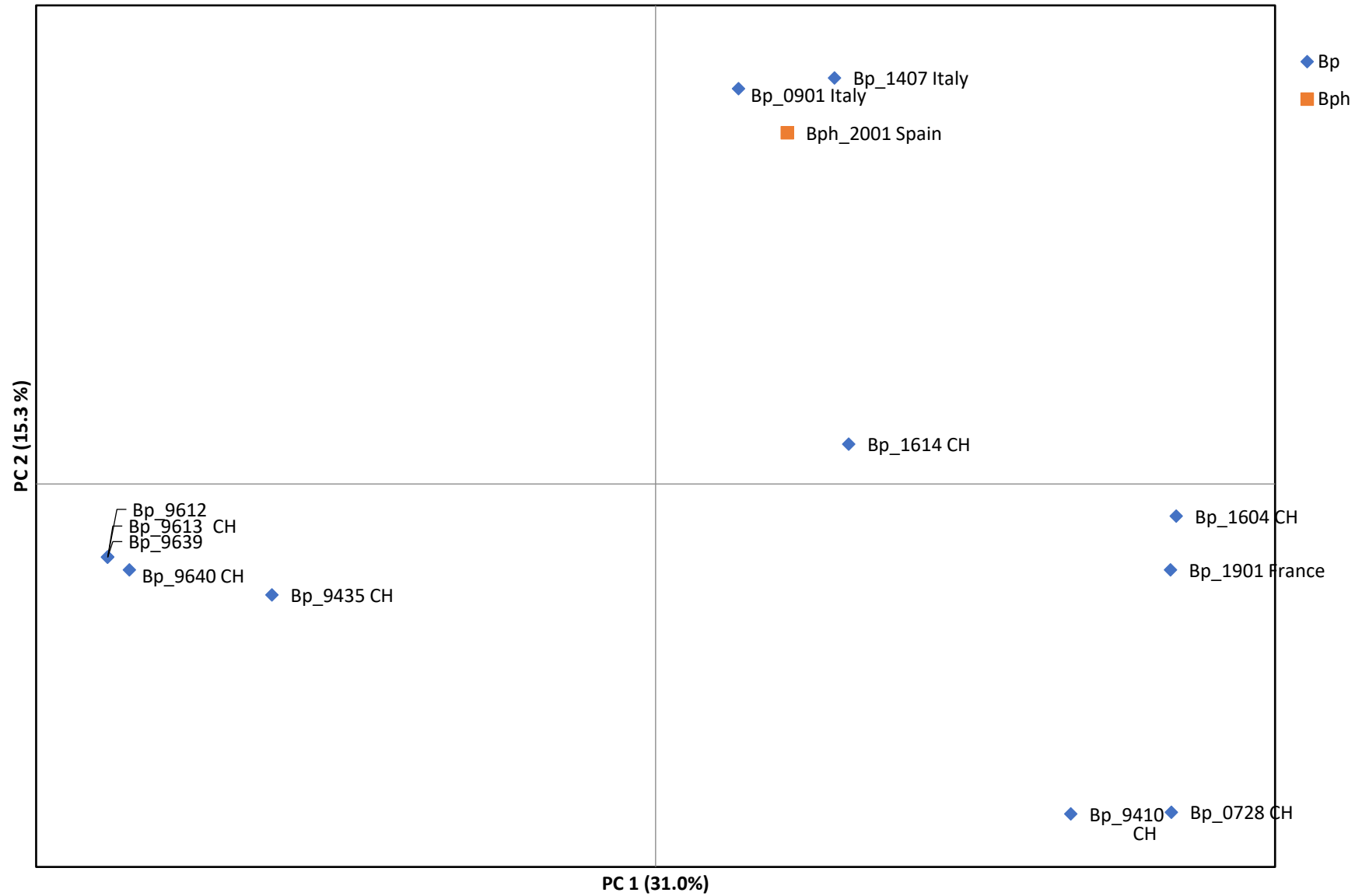

Supplement: Supplementary file 1 [file jof-08-01086-s001.zip › Suppl. S3.pdf]
